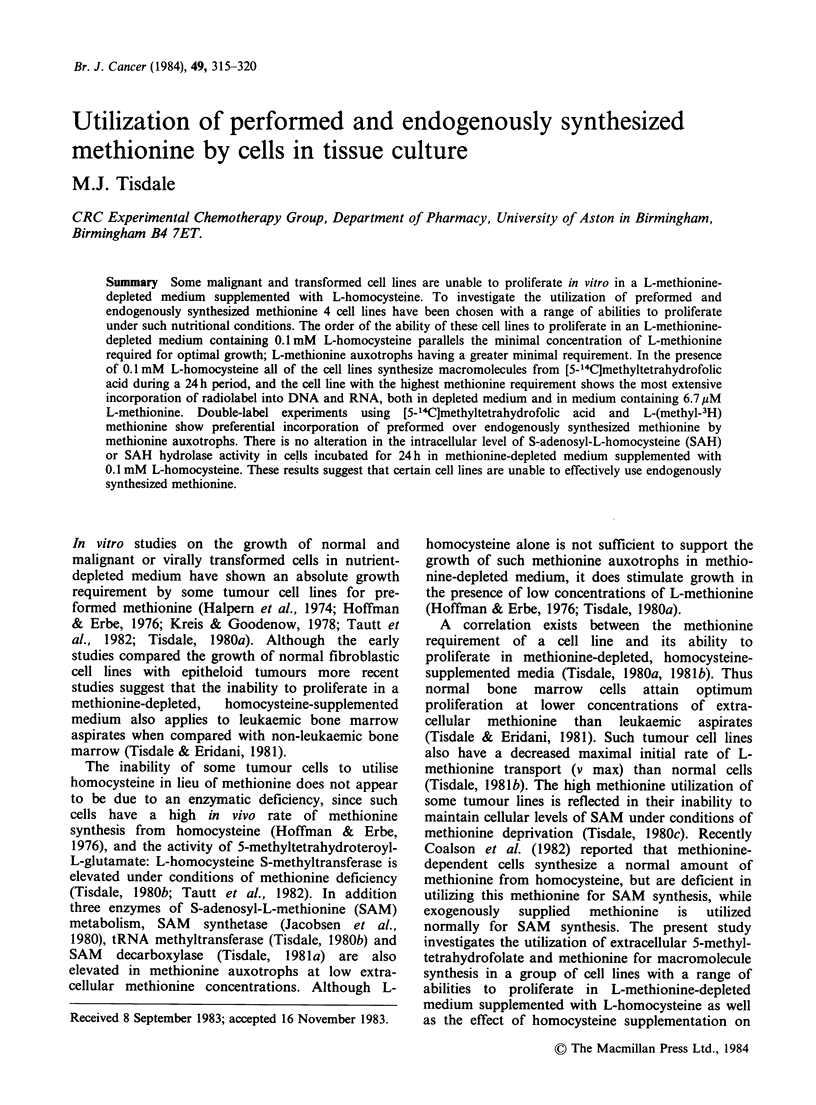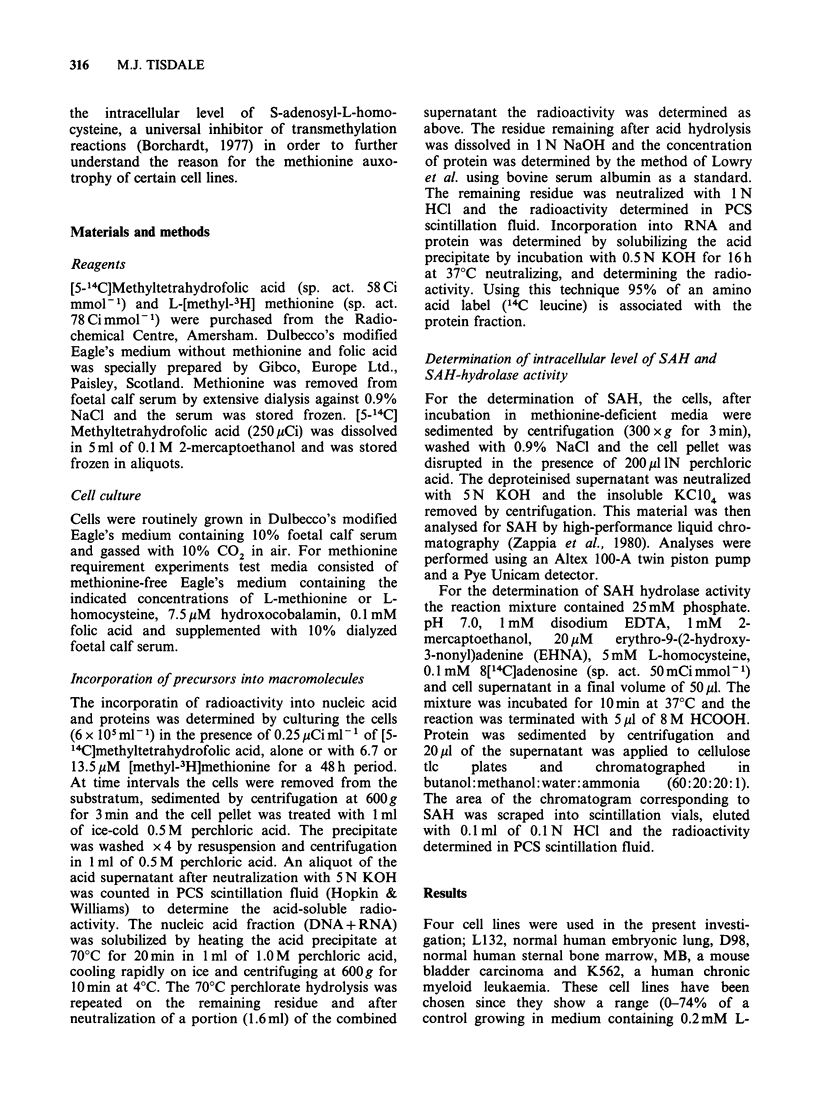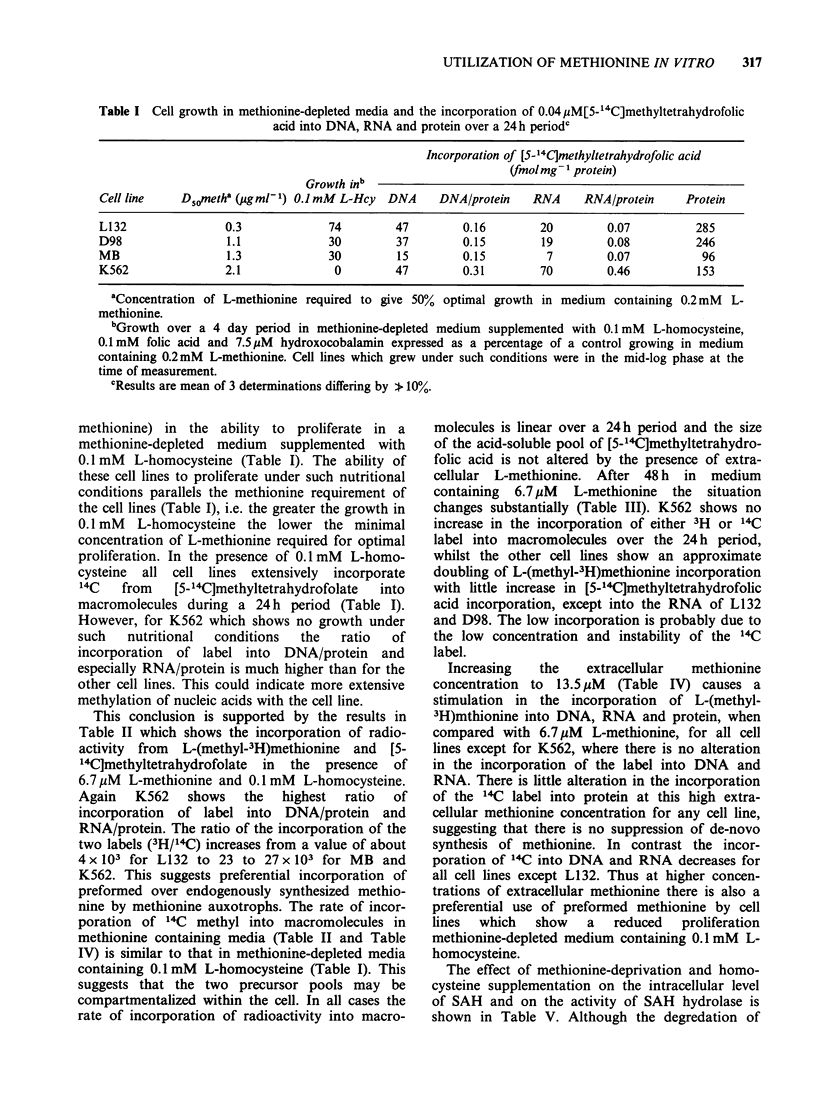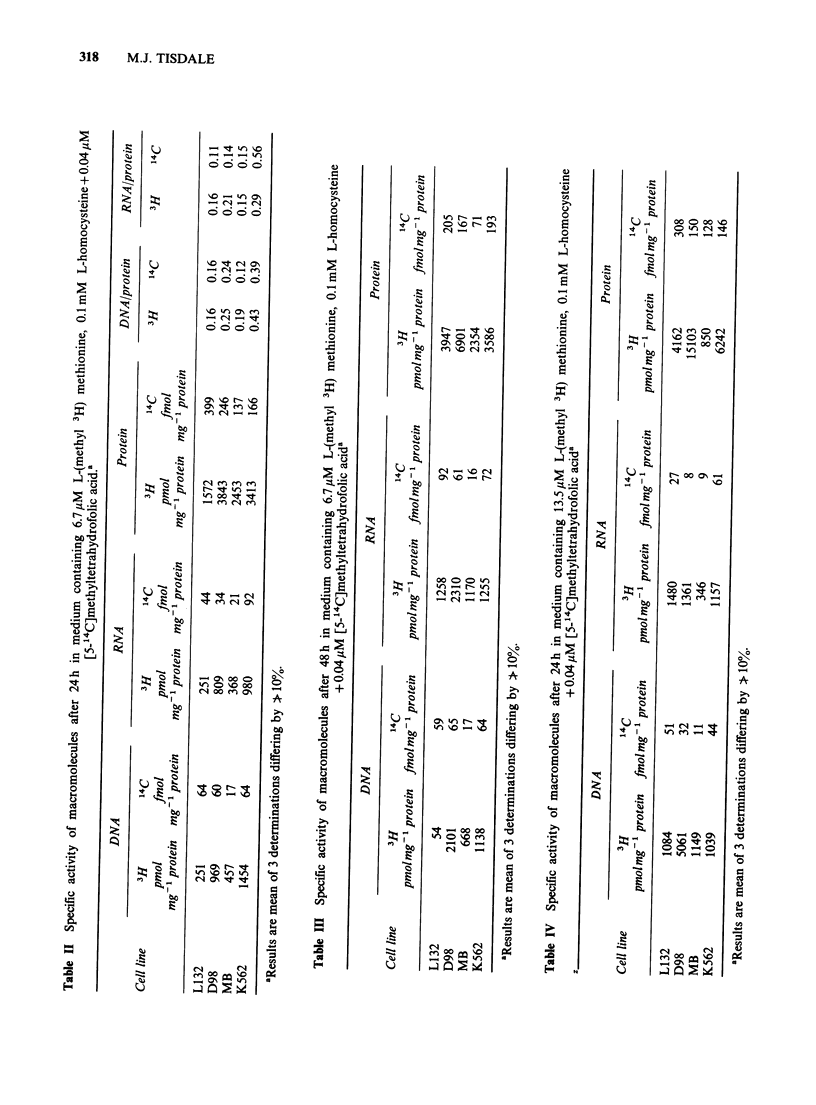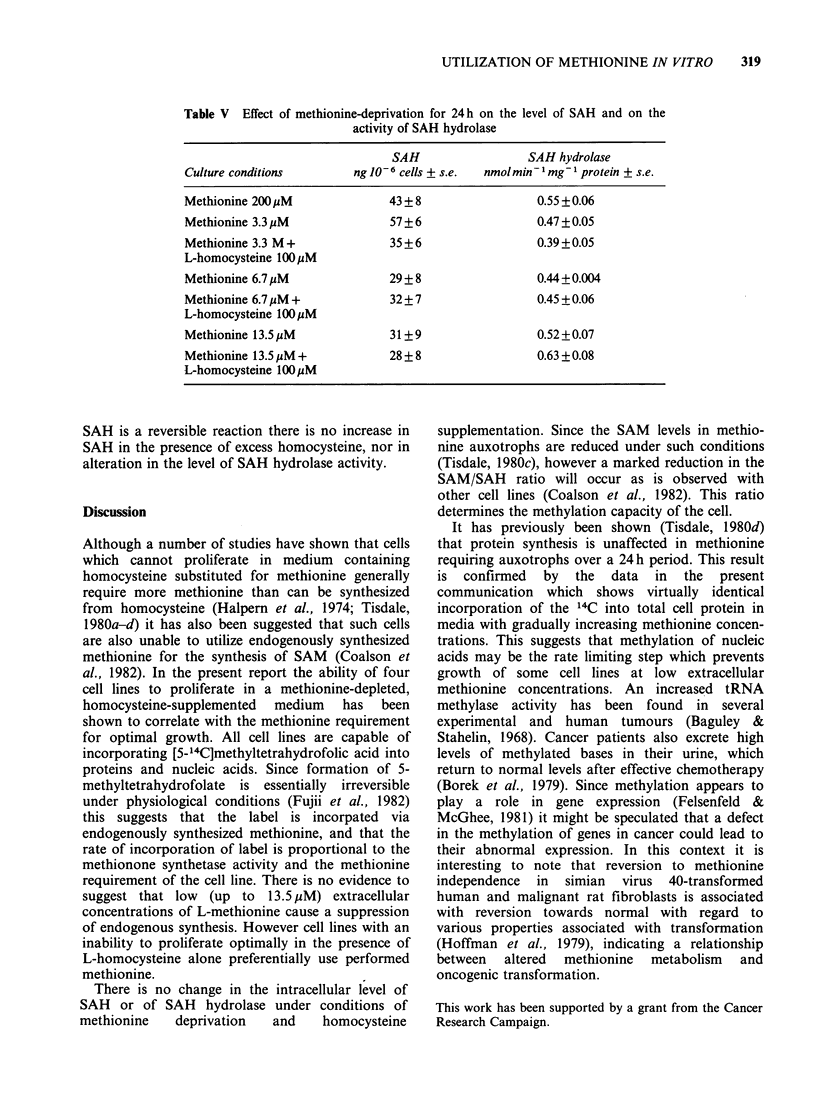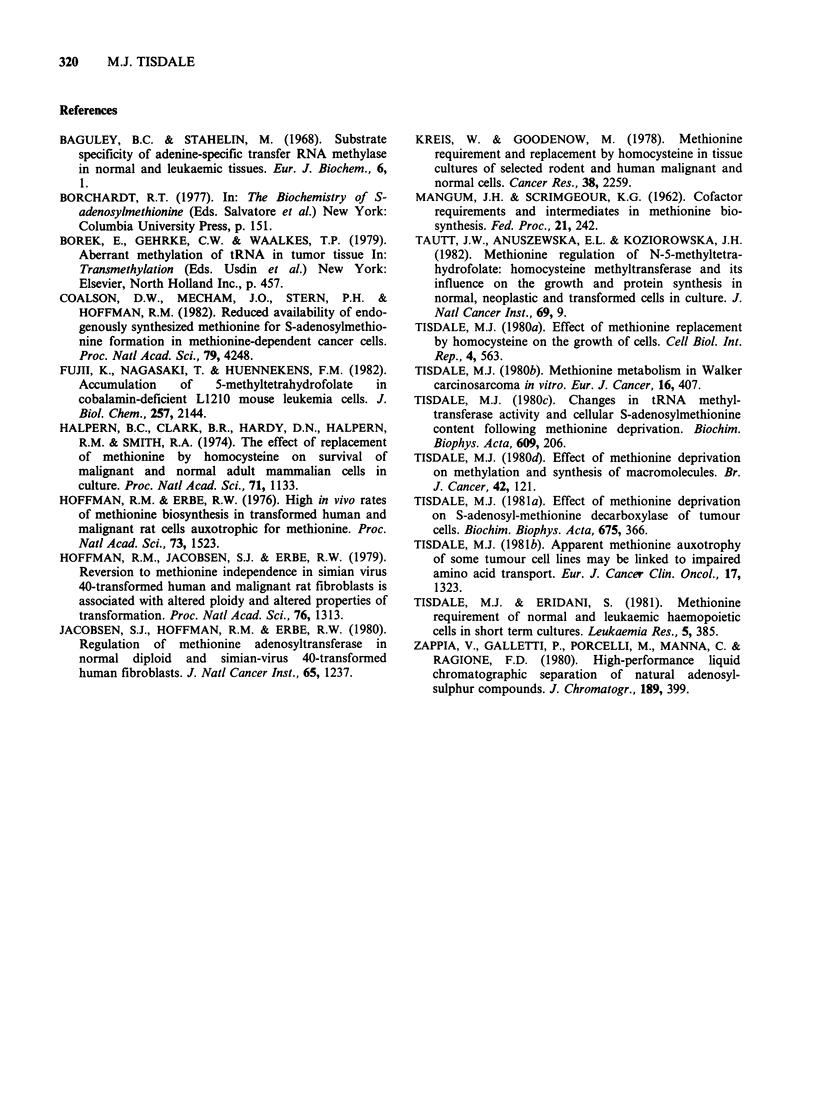# Utilization of preformed and endogenously synthesized methionine by cells in tissue culture.

**DOI:** 10.1038/bjc.1984.49

**Published:** 1984-03

**Authors:** M. J. Tisdale

## Abstract

Some malignant and transformed cell lines are unable to proliferate in vitro in a L-methionine-depleted medium supplemented with L-homocysteine. To investigate the utilization of preformed and endogenously synthesized methionine 4 cell lines have been chosen with a range of abilities to proliferate under such nutritional conditions. The order of the ability of these cell lines to proliferate in an L-methionine-depleted medium containing 0.1 mM L-homocysteine parallels the minimal concentration of L-methionine required for optimal growth; L-methionine auxotrophs having a greater minimal requirement. In the presence of 0.1 mM L-homocysteine all of the cell lines synthesize macromolecules from [5-14C]methyltetrahydrofolic acid during a 24 h period, and the cell line with the highest methionine requirement shows the most extensive incorporation of radiolabel into DNA and RNA, both in depleted medium and in medium containing 6.7 microM L-methionine. Double-label experiments using [5-14C]methyltetrahydrofolic acid and L-(methyl-3H) methionine show preferential incorporation of preformed over endogenously synthesized methionine by methionine auxotrophs. There is no alteration in the intracellular level of S-adenosyl-L-homocysteine (SAH) or SAH hydrolase activity in cells incubated for 24 h in methionine-depleted medium supplemented with 0.1 mM L-homocysteine. These results suggest that certain cell lines are unable to effectively use endogenously synthesized methionine.


Br. J. Cancer (1984), 49, 315–320

### Utilization of performed and endogenously synthesized methionine by cells in tissue culture

M.J. Tisdale

*CRC Experimental Chemotherapy Group, Department of Pharmacy, University of Aston in Birmingham, Birmingham B4 7ET.*

**Summary** Some malignant and transformed cell lines are unable to proliferate *in vitro* in a L-methionine-depleted medium supplemented with L-homocysteine. To investigate the utilization of preformed and endogenously synthesized methionine 4 cell lines have been chosen with a range of abilities to proliferate under such nutritional conditions. The order of the ability of these cell lines to proliferate in an L-methionine-depleted medium containing 0.1 mM L-homocysteine parallels the minimal concentration of L-methionine required for optimal growth; L-methionine auxotrophs having a greater minimal requirement. In the presence of 0.1 mM L-homocysteine all of the cell lines synthesize macromolecules from [5-$^{14}$C]methyltetrahydrofolic acid during a 24 h period, and the cell line with the highest methionine requirement shows the most extensive incorporation of radiolabel into DNA and RNA, both in depleted medium and in medium containing 6.7 $\mu$M L-methionine. Double-label experiments using [5-$^{14}$C]methyltetrahydrofolic acid and L-(methyl-$^{3}$H) methionine show preferential incorporation of preformed over endogenously synthesized methionine by methionine auxotrophs. There is no alteration in the intracellular level of S-adenosyl-L-homocysteine (SAH) or SAH hydrolase activity in cells incubated for 24 h in methionine-depleted medium supplemented with 0.1 mM L-homocysteine. These results suggest that certain cell lines are unable to effectively use endogenously synthesized methionine.

*In vitro* studies on the growth of normal and malignant or virally transformed cells in nutrient-depleted medium have shown an absolute growth requirement by some tumour cell lines for preformed methionine (Halpern *et al.*, 1974; Hoffman & Erbe, 1976; Kreis & Goodenow, 1978; Tautt *et al.*, 1982; Tisdale, 1980*a*). Although the early studies compared the growth of normal fibroblastic cell lines with epitheloid tumours more recent studies suggest that the inability to proliferate in a methionine-depleted, homocysteine-supplemented medium also applies to leukaemic bone marrow aspirates when compared with non-leukaemic bone marrow (Tisdale & Eridani, 1981).

The inability of some tumour cells to utilise homocysteine in lieu of methionine does not appear to be due to an enzymatic deficiency, since such cells have a high *in vivo* rate of methionine synthesis from homocysteine (Hoffman & Erbe, 1976), and the activity of 5-methyltetrahydroteroyl-L-glutamate: L-homocysteine S-methyltransferase is elevated under conditions of methionine deficiency (Tisdale, 1980*b*; Tautt *et al.*, 1982). In addition three enzymes of S-adenosyl-L-methionine (SAM) metabolism, SAM synthetase (Jacobsen *et al.*, 1980), tRNA methyltransferase (Tisdale, 1980*b*) and SAM decarboxylase (Tisdale, 1981*a*) are also elevated in methionine auxotrophs at low extra-cellular methionine concentrations. Although L-homocysteine alone is not sufficient to support the growth of such methionine auxotrophs in methionine-depleted medium, it does stimulate growth in the presence of low concentrations of L-methionine (Hoffman & Erbe, 1976; Tisdale, 1980*a*).

A correlation exists between the methionine requirement of a cell line and its ability to proliferate in methionine-depleted, homocysteine-supplemented media (Tisdale, 1980*a*, 1981*b*). Thus normal bone marrow cells attain optimum proliferation at lower concentrations of extra-cellular methionine than leukaemic aspirates (Tisdale & Eridani, 1981). Such tumour cell lines also have a decreased maximal initial rate of L-methionine transport (*v* max) than normal cells (Tisdale, 1981*b*). The high methionine utilization of some tumour lines is reflected in their inability to maintain cellular levels of SAM under conditions of methionine deprivation (Tisdale, 1980*c*). Recently Coalson *et al.* (1982) reported that methionine-dependent cells synthesize a normal amount of methionine from homocysteine, but are deficient in utilizing this methionine for SAM synthesis, while exogenously supplied methionine is utilized normally for SAM synthesis. The present study investigates the utilization of extracellular 5-methyl-tetrahydrofolate and methionine for macromolecule synthesis in a group of cell lines with a range of abilities to proliferate in L-methionine-depleted medium supplemented with L-homocysteine as well as the effect of homocysteine supplementation on

Received 8 September 1983; accepted 16 November 1983.

© The Macmillan Press Ltd., 1984

the intracellular level of S-adenosyl-L-homocysteine, a universal inhibitor of transmethylation reactions (Borchardt, 1977) in order to further understand the reason for the methionine auxotrophy of certain cell lines.

### Materials and methods

*Reagents*

[5-$^{14}$C]Methyltetrahydrofolic acid (sp. act. 58 Ci mmol$^{-1}$) and L-[methyl-$^{3}$H] methionine (sp. act. 78 Ci mmol$^{-1}$) were purchased from the Radiochemical Centre, Amersham. Dulbecco's modified Eagle's medium without methionine and folic acid was specially prepared by Gibco, Europe Ltd., Paisley, Scotland. Methionine was removed from foetal calf serum by extensive dialysis against 0.9% NaCl and the serum was stored frozen. [5-$^{14}$C] Methyltetrahydrofolic acid (250 $\mu$Ci) was dissolved in 5 ml of 0.1 M 2-mercaptoethanol and was stored frozen in aliquots.

*Cell culture*

Cells were routinely grown in Dulbecco's modified Eagle's medium containing 10% foetal calf serum and gassed with 10% $CO_2$ in air. For methionine requirement experiments test media consisted of methionine-free Eagle's medium containing the indicated concentrations of L-methionine or L-homocysteine, 7.5 $\mu$M hydroxocobalamin, 0.1 mM folic acid and supplemented with 10% dialyzed foetal calf serum.

*Incorporation of precursors into macromolecules*

The incorporatin of radioactivity into nucleic acid and proteins was determined by culturing the cells ($6 \times 10^5$ ml$^{-1}$) in the presence of 0.25 $\mu$Ci ml$^{-1}$ of [5-$^{14}$C]methyltetrahydrofolic acid, alone or with 6.7 or 13.5 $\mu$M [methyl-$^{3}$H]methionine for a 48 h period. At time intervals the cells were removed from the substratum, sedimented by centrifugation at 600 *g* for 3 min and the cell pellet was treated with 1 ml of ice-cold 0.5 M perchloric acid. The precipitate was washed ×4 by resuspension and centrifugation in 1 ml of 0.5 M perchloric acid. An aliquot of the acid supernatant after neutralization with 5 N KOH was counted in PCS scintillation fluid (Hopkin & Williams) to determine the acid-soluble radioactivity. The nucleic acid fraction (DNA + RNA) was solubilized by heating the acid precipitate at 70°C for 20 min in 1 ml of 1.0 M perchloric acid, cooling rapidly on ice and centrifuging at 600 *g* for 10 min at 4°C. The 70°C perchlorate hydrolysis was repeated on the remaining residue and after neutralization of a portion (1.6 ml) of the combined supernatant the radioactivity was determined as above. The residue remaining after acid hydrolysis was dissolved in 1 N NaOH and the concentration of protein was determined by the method of Lowry *et al.* using bovine serum albumin as a standard. The remaining residue was neutralized with 1 N HCl and the radioactivity determined in PCS scintillation fluid. Incorporation into RNA and protein was determined by solubilizing the acid precipitate by incubation with 0.5 N KOH for 16 h at 37°C neutralizing, and determining the radioactivity. Using this technique 95% of an amino acid label ($^{14}$C leucine) is associated with the protein fraction.

*Determination of intracellular level of SAH and SAH-hydrolase activity*

For the determination of SAH, the cells, after incubation in methionine-deficient media were sedimented by centrifugation (300 × *g* for 3 min), washed with 0.9% NaCl and the cell pellet was disrupted in the presence of 200 $\mu$l 1N perchloric acid. The deproteinised supernatant was neutralized with 5 N KOH and the insoluble $KC10_4$ was removed by centrifugation. This material was then analysed for SAH by high-performance liquid chromatography (Zappia *et al.*, 1980). Analyses were performed using an Altex 100-A twin piston pump and a Pye Unicam detector.

For the determination of SAH hydrolase activity the reaction mixture contained 25 mM phosphate. pH 7.0, 1 mM disodium EDTA, 1 mM 2-mercaptoethanol, 20 $\mu$M erythro-9-(2-hydroxy-3-nonyl)adenine (EHNA), 5 mM L-homocysteine, 0.1 mM 8[$^{14}$C]adenosine (sp. act. 50 mCi mmol$^{-1}$) and cell supernatant in a final volume of 50 $\mu$l. The mixture was incubated for 10 min at 37°C and the reaction was terminated with 5 $\mu$l of 8 M HCOOH. Protein was sedimented by centrifugation and 20 $\mu$l of the supernatant was applied to cellulose tlc plates and chromatographed in butanol:methanol:water:ammonia (60:20:20:1). The area of the chromatogram corresponding to SAH was scraped into scintillation vials, eluted with 0.1 ml of 0.1 N HCl and the radioactivity determined in PCS scintillation fluid.

### Results

Four cell lines were used in the present investigation; L132, normal human embryonic lung, D98, normal human sternal bone marrow, MB, a mouse bladder carcinoma and K562, a human chronic myeloid leukaemia. These cell lines have been chosen since they show a range (0–74% of a control growing in medium containing 0.2 mM L-

**Table I** Cell growth in methionine-depleted media and the incorporation of 0.04 μM[5-$^{14}$C]methyltetrahydrofolic acid into DNA, RNA and protein over a 24 h period[c]

| Cell line | $D_{50}$meth[a] (μg ml$^{-1}$) | Growth in[b] 0.1 mM L-Hcy | Incorporation of [5-$^{14}$C]methyltetrahydrofolic acid (fmol mg$^{-1}$ protein): DNA | DNA/protein | RNA | RNA/protein | Protein |
|---|---|---|---|---|---|---|---|
| L132 | 0.3 | 74 | 47 | 0.16 | 20 | 0.07 | 285 |
| D98 | 1.1 | 30 | 37 | 0.15 | 19 | 0.08 | 246 |
| MB | 1.3 | 30 | 15 | 0.15 | 7 | 0.07 | 96 |
| K562 | 2.1 | 0 | 47 | 0.31 | 70 | 0.46 | 153 |

[a]Concentration of L-methionine required to give 50% optimal growth in medium containing 0.2 mM L-methionine.

[b]Growth over a 4 day period in methionine-depleted medium supplemented with 0.1 mM L-homocysteine, 0.1 mM folic acid and 7.5 μM hydroxocobalamin expressed as a percentage of a control growing in medium containing 0.2 mM L-methionine. Cell lines which grew under such conditions were in the mid-log phase at the time of measurement.

[c]Results are mean of 3 determinations differing by $\not>$10%.

methionine) in the ability to proliferate in a methionine-depleted medium supplemented with 0.1 mM L-homocysteine (Table I). The ability of these cell lines to proliferate under such nutritional conditions parallels the methionine requirement of the cell lines (Table I), i.e. the greater the growth in 0.1 mM L-homocysteine the lower the minimal concentration of L-methionine required for optimal proliferation. In the presence of 0.1 mM L-homocysteine all cell lines extensively incorporate $^{14}$C from [5-$^{14}$C]methyltetrahydrofolate into macromolecules during a 24 h period (Table I). However, for K562 which shows no growth under such nutritional conditions the ratio of incorporation of label into DNA/protein and especially RNA/protein is much higher than for the other cell lines. This could indicate more extensive methylation of nucleic acids with the cell line.

This conclusion is supported by the results in Table II which shows the incorporation of radioactivity from L-(methyl-$^3$H)methionine and [5-$^{14}$C]methyltetrahydrofolate in the presence of 6.7 μM L-methionine and 0.1 mM L-homocysteine. Again K562 shows the highest ratio of incorporation of label into DNA/protein and RNA/protein. The ratio of the incorporation of the two labels ($^3$H/$^{14}$C) increases from a value of about $4 \times 10^3$ for L132 to 23 to $27 \times 10^3$ for MB and K562. This suggests preferential incorporation of preformed over endogenously synthesized methionine by methionine auxotrophs. The rate of incorporation of $^{14}$C methyl into macromolecules in methionine containing media (Table II and Table IV) is similar to that in methionine-depleted media containing 0.1 mM L-homocysteine (Table I). This suggests that the two precursor pools may be compartmentalized within the cell. In all cases the rate of incorporation of radioactivity into macromolecules is linear over a 24 h period and the size of the acid-soluble pool of [5-$^{14}$C]methyltetrahydrofolic acid is not altered by the presence of extracellular L-methionine. After 48 h in medium containing 6.7 μM L-methionine the situation changes substantially (Table III). K562 shows no increase in the incorporation of either $^3$H or $^{14}$C label into macromolecules over the 24 h period, whilst the other cell lines show an approximate doubling of L-(methyl-$^3$H)methionine incorporation with little increase in [5-$^{14}$C]methyltetrahydrofolic acid incorporation, except into the RNA of L132 and D98. The low incorporation is probably due to the low concentration and instability of the $^{14}$C label.

Increasing the extracellular methionine concentration to 13.5 μM (Table IV) causes a stimulation in the incorporation of L-(methyl-$^3$H)mthionine into DNA, RNA and protein, when compared with 6.7 μM L-methionine, for all cell lines except for K562, where there is no alteration in the incorporation of the label into DNA and RNA. There is little alteration in the incorporation of the $^{14}$C label into protein at this high extracellular methionine concentration for any cell line, suggesting that there is no suppression of de-novo synthesis of methionine. In contrast the incorporation of $^{14}$C into DNA and RNA decreases for all cell lines except L132. Thus at higher concentrations of extracellular methionine there is also a preferential use of preformed methionine by cell lines which show a reduced proliferation methionine-depleted medium containing 0.1 mM L-homocysteine.

The effect of methionine-deprivation and homocysteine supplementation on the intracellular level of SAH and on the activity of SAH hydrolase is shown in Table V. Although the degredation of

**Table V** Effect of methionine-deprivation for 24 h on the level of SAH and on the activity of SAH hydrolase

| *Culture conditions* | *SAH* $ng\ 10^{-6}$ *cells* ± *s.e.* | *SAH hydrolase* $nmol\ min^{-1}\ mg^{-1}$ *protein* ± *s.e.* |
|---|---|---|
| Methionine 200 μM | 43 ± 8 | 0.55 ± 0.06 |
| Methionine 3.3 μM | 57 ± 6 | 0.47 ± 0.05 |
| Methionine 3.3 M + L-homocysteine 100 μM | 35 ± 6 | 0.39 ± 0.05 |
| Methionine 6.7 μM | 29 ± 8 | 0.44 ± 0.004 |
| Methionine 6.7 μM + L-homocysteine 100 μM | 32 ± 7 | 0.45 ± 0.06 |
| Methionine 13.5 μM | 31 ± 9 | 0.52 ± 0.07 |
| Methionine 13.5 μM + L-homocysteine 100 μM | 28 ± 8 | 0.63 ± 0.08 |

SAH is a reversible reaction there is no increase in SAH in the presence of excess homocysteine, nor in alteration in the level of SAH hydrolase activity.

### Discussion

Although a number of studies have shown that cells which cannot proliferate in medium containing homocysteine substituted for methionine generally require more methionine than can be synthesized from homocysteine (Halpern *et al.*, 1974; Tisdale, 1980*a–d*) it has also been suggested that such cells are also unable to utilize endogenously synthesized methionine for the synthesis of SAM (Coalson *et al.*, 1982). In the present report the ability of four cell lines to proliferate in a methionine-depleted, homocysteine-supplemented medium has been shown to correlate with the methionine requirement for optimal growth. All cell lines are capable of incorporating [5-$^{14}$C]methyltetrahydrofolic acid into proteins and nucleic acids. Since formation of 5-methyltetrahydrofolate is essentially irreversible under physiological conditions (Fujii *et al.*, 1982) this suggests that the label is incorpated via endogenously synthesized methionine, and that the rate of incorporation of label is proportional to the methionone synthetase activity and the methionine requirement of the cell line. There is no evidence to suggest that low (up to 13.5 μM) extracellular concentrations of L-methionine cause a suppression of endogenous synthesis. However cell lines with an inability to proliferate optimally in the presence of L-homocysteine alone preferentially use performed methionine.

There is no change in the intracellular level of SAH or of SAH hydrolase under conditions of methionine deprivation and homocysteine supplementation. Since the SAM levels in methionine auxotrophs are reduced under such conditions (Tisdale, 1980*c*), however a marked reduction in the SAM/SAH ratio will occur as is observed with other cell lines (Coalson *et al.*, 1982). This ratio determines the methylation capacity of the cell.

It has previously been shown (Tisdale, 1980*d*) that protein synthesis is unaffected in methionine requiring auxotrophs over a 24 h period. This result is confirmed by the data in the present communication which shows virtually identical incorporation of the $^{14}$C into total cell protein in media with gradually increasing methionine concentrations. This suggests that methylation of nucleic acids may be the rate limiting step which prevents growth of some cell lines at low extracellular methionine concentrations. An increased tRNA methylase activity has been found in several experimental and human tumours (Baguley & Stahelin, 1968). Cancer patients also excrete high levels of methylated bases in their urine, which return to normal levels after effective chemotherapy (Borek *et al.*, 1979). Since methylation appears to play a role in gene expression (Felsenfeld & McGhee, 1981) it might be speculated that a defect in the methylation of genes in cancer could lead to their abnormal expression. In this context it is interesting to note that reversion to methionine independence in simian virus 40-transformed human and malignant rat fibroblasts is associated with reversion towards normal with regard to various properties associated with transformation (Hoffman *et al.*, 1979), indicating a relationship between altered methionine metabolism and oncogenic transformation.

This work has been supported by a grant from the Cancer Research Campaign.

**References**

BAGULEY, B.C. & STAHELIN, M. (1968). Substrate specificity of adenine-specific transfer RNA methylase in normal and leukaemic tissues. *Eur. J. Biochem.*, **6**, 1.

BORCHARDT, R.T. (1977). In: *The Biochemistry of S-adenosylmethionine* (Eds. Salvatore *et al.*) New York: Columbia University Press, p. 151.

BOREK, E., GEHRKE, C.W. & WAALKES, T.P. (1979). Aberrant methylation of tRNA in tumor tissue In: *Transmethylation* (Eds. Usdin *et al.*) New York: Elsevier, North Holland Inc., p. 457.

COALSON, D.W., MECHAM, J.O., STERN, P.H. & HOFFMAN, R.M. (1982). Reduced availability of endogenously synthesized methionine for S-adenosylmethionine formation in methionine-dependent cancer cells. *Proc. Natl Acad. Sci.*, **79**, 4248.

FUJII, K., NAGASAKI, T. & HUENNEKENS, F.M. (1982). Accumulation of 5-methyltetrahydrofolate in cobalamin-deficient L1210 mouse leukemia cells. *J. Biol. Chem.*, **257**, 2144.

HALPERN, B.C., CLARK, B.R., HARDY, D.N., HALPERN, R.M. & SMITH, R.A. (1974). The effect of replacement of methionine by homocysteine on survival of malignant and normal adult mammalian cells in culture. *Proc. Natl Acad. Sci.*, **71**, 1133.

HOFFMAN, R.M. & ERBE, R.W. (1976). High *in vivo* rates of methionine biosynthesis in transformed human and malignant rat cells auxotrophic for methionine. *Proc. Natl Acad. Sci.*, **73**, 1523.

HOFFMAN, R.M., JACOBSEN, S.J. & ERBE, R.W. (1979). Reversion to methionine independence in simian virus 40-transformed human and malignant rat fibroblasts is associated with altered ploidy and altered properties of transformation. *Proc. Natl Acad. Sci.*, **76**, 1313.

JACOBSEN, S.J., HOFFMAN, R.M. & ERBE, R.W. (1980). Regulation of methionine adenosyltransferase in normal diploid and simian-virus 40-transformed human fibroblasts. *J. Natl Cancer Inst.*, **65**, 1237.

KREIS, W. & GOODENOW, M. (1978). Methionine requirement and replacement by homocysteine in tissue cultures of selected rodent and human malignant and normal cells. *Cancer Res.*, **38**, 2259.

MANGUM, J.H. & SCRIMGEOUR, K.G. (1962). Cofactor requirements and intermediates in methionine biosynthesis. *Fed. Proc.*, **21**, 242.

TAUTT, J.W., ANUSZEWSKA, E.L. & KOZIOROWSKA, J.H. (1982). Methionine regulation of N-5-methyltetrahydrofolate: homocysteine methyltransferase and its influence on the growth and protein synthesis in normal, neoplastic and transformed cells in culture. *J. Natl Cancer Inst.*, **69**, 9.

TISDALE, M.J. (1980*a*). Effect of methionine replacement by homocysteine on the growth of cells. *Cell Biol. Int. Rep.*, **4**, 563.

TISDALE, M.J. (1980*b*). Methionine metabolism in Walker carcinosarcoma *in vitro*. *Eur. J. Cancer*, **16**, 407.

TISDALE, M.J. (1980*c*). Changes in tRNA methyltransferase activity and cellular S-adenosylmethionine content following methionine deprivation. *Biochim. Biophys. Acta*, **609**, 206.

TISDALE, M.J. (1980*d*). Effect of methionine deprivation on methylation and synthesis of macromolecules. *Br. J. Cancer*, **42**, 121.

TISDALE, M.J. (1981*a*). Effect of methionine deprivation on S-adenosyl-methionine decarboxylase of tumour cells. *Biochim. Biophys. Acta*, **675**, 366.

TISDALE, M.J. (1981*b*). Apparent methionine auxotrophy of some tumour cell lines may be linked to impaired amino acid transport. *Eur. J. Cancer Clin. Oncol.*, **17**, 1323.

TISDALE, M.J. & ERIDANI, S. (1981). Methionine requirement of normal and leukaemic haemopoietic cells in short term cultures. *Leukaemia Res.*, **5**, 385.

ZAPPIA, V., GALLETTI, P., PORCELLI, M., MANNA, C. & RAGIONE, F.D. (1980). High-performance liquid chromatographic separation of natural adenosylsulphur compounds. *J. Chromatogr.*, **189**, 399.